# Tubercular Nodules of the Cerebellum

**Published:** 1889-03

**Authors:** William C. Krauss

**Affiliations:** Attica, N. Y.; 5 Rue Rollin, Paris


### *TUBERCULAR NODULES OF THE CEREBELLUM.*

By WILLIAM C. KRAUSS, B. S., M. D., Attica, N. Y.

Extracts from an Inaugural Dissertation delivered to the Faculty of the Friedrich-Wilhelm's Universität, for the degree Doctor of Medicine. Publicly defended, Aug. 8, 1888.

The history of the case, the examination of which forms the basis of this dissertation, is as follows :

In January, 1888, Willy L., a child of two years, appeared for the first time in the polyclinic for nervous diseases of Professors Mendel and Eulenburg (Berlin).

The child was emaciated, thin, pale, and showed in general a scrofulous disposition. The father had always been a healthy man. The mother, with the exception of being an alcoholic, had always enjoyed good health. The mother relates the following history :

For some time past, the child has been subject to attacks, regular in appearance, occurring every five minutes. During these attacks, which may be regarded as epileptiform, clonic contractions appear on the right side, particularly involving the right arm, the head is drawn backwards and is rigid. During these paroxysms, the patient is not in an unconscious condition, nevertheless the mental faculties are somewhat blunted. Movements of the orbits, general convulsions, tonic and clonic, and paresis of the sphincters are wanting. The mother reports that the child complains frequently of headaches, during the night is restless, and sleep is much disturbed. During the past week, the child has become quiet and apathetic. The mother also relates that four children have died of similar attacks.

*Status Præsens.*—The child is in a state of apathy, paying no attention to surrounding objects; is indifferent during the examination. The head is easily moved; no rigidity at the back of the neck. The movements of the eyeballs are normal, the pupils react readily and show no difference in their size. An examination of the fundus of the eye was not undertaken. The tongue is protruded easily, shows no deviation, shows no appearance of scars, either recent or former. The face shows no dissimilarity of the two sides. An examination of the extremities reveals no paresis or paralysis. The general sensibility is not affected.

The child, in attempting to walk, shows a staggering gait with a tendency to fall backwards, so that it is impossible to make any progress without support. The reflexes are all normal.

An examination of the lungs shows consolidation in both upper lobes, with fine bronchial râles.

The heart sounds are distinct; no murmurs appreciable. The pulse is not retarded. The examination of the remaining organs gives a negative result.

The diagnosis is that of an affection of the brain—resp. cerebellum, based upon the epileptiform convulsions, headaches, staggering gait, dizziness, nocturnal cries and semi-comatose condition. With reference to the age, constitution and condition of the lungs, it is plausible to infer that the affection is of a tubercular nature.

The child was presented in the clinic of Professor Mendel, and a diagnosis of tuberculosis of the cerebellum was made.

Ten days later, exitus letalis occurred. The autopsy, made under the supervision of Professor Mendel, verified the diagnosis.

The report of the autopsy is as follows:

The body, gracile, emaciated; shows a slight amount of rigor mortis. The cranium is easily removed; no adhesions of the dura. The dura presents nothing of interest. The pia is clear, easily removed; no adhesions to the cortex. Blood-vessels are not engorged.

In attempting to remove the brain from the cranium, the left hemisphere of the cerebellum offers resistance, being firmly adherent to the dura. By using some force, the adhesions are overcome, but there remains attached to the dura round, somewhat regular mass, about the size of a hazelnut. The left hemisphere of the cerebellum shows a cavity with irregular walls, corresponding to the tumor attached to the dura.

The cerebrum presents a smooth surface, with no perceptible resistance. Cross sections reveal nothing abnormal. The surface of the cerebellum shows, with the exception of the cavity above referred to, nothing abnormal. A closer examination, however, reveals distinctly the presence of six round hard tumors, the size varying from a bean to a hazelnut, and a number of smaller ones less distinct, in the substance of the cerebellum. Cross sections show the tumors distribbuted in the superior and inferior vermiform processes, and the inferior lobes of the right and left hemispheres.

A section of the tumors shows them to be grayish-white, of a distinct concentric formation. The center shows a soft degenerated caseous mass. The surrounding tissues are edematous.

The examination of the thoracic cavity reveals the pleura pulmonalis covered with numerous yellowish points, about the size of a pin's head. Sections through the lungs reveal in great number the same deposits. The mediastinal glands are swollen, and present the same caseous consistency as the tumors in the cerebellum. The heart shows nothing abnormal.

By request of the parents, the examination was limited to the cranial and thoracic cavities.

Anatomical diagnosis: Miliary tuberculosis, especially characterized by the formation of tubercular deposits in the cerebellum.

The cerebellum was immediately placed in a seventy-five per cent. solution of alcohol, seven days later in ninety-five per cent., and allowed to remain in the latter six weeks; small pieces of the lungs, and the mediastinal glands, were treated in the same manner. After sufficient hardening, the pieces were imbedded in celloidin, and sections made as thin as possible.

The nodule, to which I will pay special attention, is a tumor having the size and form of a hazelnut, with a diameter of ten millimeters. It is situated in the inferior-posterior lobe of the right hemisphere, occupying the greater portion of this lobe. A longitudinal section through cerebellum and nodule, shows it to extend upwards into the region of the corpus dentatum. The grayish coloration of its external layers permit it to be easily separated from its surrounding tissue, while its center presents two large opaque caseous masses, surrounded by an amorphous structure, not possessing the caseous character of the former, being less opaque and of a greater consistency. The central masses are surrounded by a zone, having a concentric laminated structure of a light gray color. The transition into the normal tissues of the cerebellum is not a sharp one, the gray coloration of the external zone of the nodule gradually disappearing. During the section-cutting, the nodule shows the following microscopic changes: Gradually diminishing in size, and in the center several small opaque, irregular masses appear. Laterally, another group of small nodules appear, increasing in size gradually; conglomeration forming a new nodule laterally, and posterior to the one first described. The lobus inferior-posterior of the right hemisphere, therefore, possesses two tumors of about equal size: the one situated mesad and anteriorly, the other laterally and posteriorly.

The microscopic examination was limited to the structure of the nodules, the changes having taken place in the surrounding tissues, and the presence of the tuberkel-bacilus. The staining methods used were the ammoniac-carmine, picro-carmine, alum-carmine, Læmatoxylin and Niessl's; for the bacilus, the Koch-Ehrlich, Ziehl-Neelsen, Gram and Carbolfuchsine. The relation of the nodule to the surrounding normal tissue was striking, in that the stain was much deeper, and, therefore, could be sharply differentiated. In the nodule itself, the stain was irregular. The caseous degenerated masses were intensely colored, while the internodular tissues, as well as the external zone, were less intense.

The bulk of the nodule is formed by the caseous degenerated masses, surrounded by a zone rich in cells. In these masses, no structure or organization is to be found. They are composed of a compact granular material, possessing, here and there, the atrophied walls of blood-vessels which have not yet undergone degeneration. These masses, which must be considered as the detritus of degenerated elements, are products of a caseous metamorphosis, produced by the rapid downfall of cellular elements. They resemble closely the tubercular-like masses which arise in the metamorphosis of carcinoma and other processes. These masses were formerly considered as characteristic of tuberculosis. Lebert and Bayle held this opinion, and thought that genuine tubercular deposits were present in carcinoma. Others held that these deposits were the analogues of an exudation. Von Bischoff considered the blood as the origin of the tubercle formation. . . .

Mauthner was also, at that time, of the same opinion; Virchow, however, showed that these masses, as they appeared in the metamorphosis of cancers, were only the accumulation of downfallen and necrosed carcinomatous elements. He considers the real tubercle as a granular mass, with a cellular organization. Its constituents are produced by the proliferation of preëxisting elements, which later on perish, necrose, thicken and undergo the same tubercular caseous metamorphosis which takes place in carcinomas and abscesses. He calls particular attention to the fact that the tubercular metamorphosis is not the characteristic of a specific process—of a particular constitution. (This assertion was made prior to the discovery of the tuberkel-bacilus. The degenerative process remains, however, unaltered, the specific character of the process being dependent upon the bacillus.)

" Tubercularization, or tubercular metamorphosis, consists really in the transformation of old as well as newly formed elements, whereby the abolition of nutritive and generative forces produces a mortification, necrosis of the tissues, which, later on, undergo a resorption of their fluid contents and thickening of those parts devoid of nutrition "

In the tissue between the caseous masses, transverse and longitudinal sections of blood-vessels are still recognizable. The walls of the same show marked hypertrophy, so that the lumen is entirely obliterated. Shreds of connective tissue-fibers are also present, showing, however, no particular organization. Cells of different sizes, especially the small round cells, are everywhere abundant in this tissue; giant cells, however, could not be found.

The large cheesy masses were formerly considered as a single tubercle, and the presence of a single mass in the brain was denominated a solitary tubercle. In reality these masses are formed by the conglomeration of many primary tubercles. Baillie was the first who called attention to the fact, that the large single tubercular nodules are the results of the conglomeration of many miliary tubercles. Microscopically one sees, in the external zone of the nodule, many small dark points closely crowded together, forming a layer around the cheesy masses. A closer examination shows these points to be nests of round cells, concentrically arranged. Between these cells a fine network of connective tissue formation is seen. The center of these primary tubercles are at first clear, transcendant, but later on take a decidedly darker stain. A degeneration and necrosis of the cellular elements commences at the center and propagates itself towards the periphery. These nests of cells are the true tubercles which Virchow calls miliary--more accurately submiliary. Through the conglomeration of these miliary herds, which always appear at the periphery of the original herd, the growth of the same is explained. In other words, the growth of the nodule is explained by the opposition of new miliary tubercles at the periphery, and not through primary degenerative changes at the center radiating towards the periphery. In the posterior part of the lobe inferior-posterior, many such nests were found closely pressed together, some which did not show an opaque mass at the center, others in which the centers had already undergone the cheesy degeneration. The substance between these nests is rich in cells, but non-vascular. The serial section reveals these masses becoming more and more opaque, the masses confluent, the appearance of new tubercles at the periphery gradually increasing the size until the new nodule is distinctly appreciable. . . .

The large giant cells with numerous nuclei which Langhaus and others have frequently found in tubercular masses, were not detected. Bergkammer recently described a case in which he examined tubercular nodules of the liver, spleen, kidneys, and bronchial glands, without having found the giant cells.

The outer zone of the nodule consists of two concentric layers of granular cells, which are easily distinguishable from each other, the inner layer containing a large number of cells, the outer layer comparatively barren. The latter borders on and terminates in the normal tissues of the cerebellum. What particularly characterizes these layers are their granular cells, connective tissue fibers and blood-vessels.

The inner layer is composed chiefly of small granular cells closely crowded together, and resemble the granular cells of the cerebellum. The larger part consists of small round cells of ca. six to seven micromillimeters diameter. They possess a large granular nucleus, occupying the greater part of the cell body. Occasionally, large oval cells with a longitudinal diameter of seventeen to twenty micromillimeters and a transverse diameter of nine to ten micromillimeters are to be seen. They also possess a large granular nucleus of seven micromillimeters diameter, which by their darker strain are easily distinguishable from the cell body. Other cells of various sizes are also present, in which no nucleus is discernible, the whole cell having an opaque granular appearance. These cells are quite regularly distributed, have no tendency to group themselves into masses, and differ from the granular cells of the cerebellum by taking a lighter stain and by showing a great variance in their form and size.

A second constituent of this layer is a dense new-growth of connective tissue fibers. These fibers, which are interlaced between the cells, are spindle-shaped, and in this stage of their development exist more or less disconnected. A complete connective tissue formation, encapsuling the nodule, was not present.

The inner layer appears to form macroscopically the external zone of the nodule; nevertheless, it is necessary to distinguish an external layer which marks the transition from nodule into normal tissues. In this layer, granular cells possessing a diameter of six to seven micromillimeters and a large granular nucleus are recognizable. These cells are comparatively few in number, and diminish as one approaches the normal cerebellar tissues, so that in some places it is difficult to separate the normal from the abnormal tissues.

A degeneration or destruction of nerve fibers is not perceptible in this outer layer, but in the inner layer the formation of connective tissue fibers bespeaks the downfall of nervous elements.

The condition of the blood-vessels in the two layers, especially in the outer one, shows important and interesting pathological changes. In the first place, the vessels are more numerous than in the surrounding tissues, and accept a much deeper stain than the normal vessels. The lumen is greatly diminished, and in some places almost obliterated. In other places, a spindle-shaped dilatation, resembling somewhat imperfectly an aneurismal growth is to be found. The walls themselves are also implicated. The intima is in many places the seat of an ulcerative process, destroying the sharp contours of the same. The adventitia shows marked hypertrophy, and has a wavy appearance. The walls also present an increase in the number of their cells, being particularly numerous in the middle layers of the adventitia. These cells are round, small, in some places grouped together, in other places isolated.

No trace of blood extravasation, diapedesis of the blood corpuscles, embolic or thrombotic changes could be detected either in the nodule and its surroundings or in the normal tissues of the cerebellum. The vessels of the pia and cerebellum presented a normal appearance.

The neighboring tissues, although in the first state edematous, showed microscopically only an increase in the number of their cells. These cells proved to be small round granular cells, similar to those of the nuclear layer of the cerebellum, and were especially abundant near the corpus dentatum, gradually increasing in number towards the nodule.

Other changes in the cerebellum were not detected.

Tubercular tumors of the cerebellum, as they appear so frequently in children, offer, anatomically, no peculiarities which characterize them from other tubercular growths in other organs of the body. Here their structure as well as their growth are similar to those which appear in the choroid membrane, liver, lungs, spleen, etc. Organs which possess a high state of vascularization, and where the lymphatic system is well developed, are the favorite places for the development of tubercles. The lungs, liver, spleen, brain, and intestines, are most frequently the seat of miliary eruptions.

In the cerebrum they may take their origin from the vessels proper, or from the vessels of the pia, and grow into the surrounding tissues. Generally they choose the transition substance between the white and gray matter, but may be found in all parts of the cerebrum and cerebellum. In the cerebellum they have been found in the upper and lower vermiform processes, in the hemispheres, and even in the crura cerebelli ad pontem. In those cases where tubercular nodules are present in the cerebellum, other nodules may be found co-existing in various regions of the hemispheres-pons, crura cerebri, optic thalamus, corpora striata, and less frequently in the quadrigemina and crural cerebelli. As Virchow has remarked, their favorite seat is in the highly vascular layers of the gray tissues, either of the cortex or centers; from here they invade the white substance, but only secondarily.

The frequency of their appearance in the cerebellum has been evidenced by many compilations already made. According to Steffen, their presence in the cerebellum, as compared with their presence in all other regions of the cerebrum combined, bears a ratio of one to four. Barthez and Rilliet, on the other hand, found but a very slight difference. Recently, Pribram has made compilations to test this question, and found in sixty-seven cases of simple tuberculosis of the encephalon, sixteen cases in which the cerebellum alone was the seat of the affection. In 136 cases of multiple herds, the cerebellum was affected thirty-eight times. From his results, the cerebellum is found to be the seat of tubercular nodules in twenty-five per cent. of all cases. Birch-Hirschfeld found, in 108 cases, the cerebellum affected thirty-six times, or a ratio of one to three. The results of Pribram's compilation agree with those of Steffen, while those of Birch-Hirschfeld and Barthez and Rilliet disagree.

Although tuberculosis of the cerebellum is a common adjunct of miliary tuberculosis, deposits of miliary tubercles on the pia are absent in the great majority of cases. Barthez and Rilliet, in a great number of cases which came under their observation, found these deposits but twice. Steffen, on the contrary, found that miliary deposits were a common occurrence on the pia of the cerebellum, as well as on the convexity of the cerebrum.

*Localization.*—The question as to the precise localization in the cerebellum, whether the seat of the nodules is more common in the one than in the other hemisphere, has called forth many opinions and statistics.

Cubasch, analyzing eighty-six cases, found the nodules—

| | |
|---|---|
| In the right hemisphere . . . . . . . . . . . . . | 29 times. |
| In the left hemisphere . . . . . . . . . . . . . | 19 " |
| In both hemispheres at the same time . . . . . . | 16 " |
| In the middle lobes and vermiform processes . . | 9 " |
| Not stated . . . . . . . . . . . . . . . . . . | 13 " |

The result of my analysis agrees with that of Cubasch, in that the right hemisphere is found to be more often the seat of the disease than the left hemisphere. Out of thirty-six cases which I have been able to gather from recent literature, I found the nodules—

| | |
|---|---|
| In the right hemisphere . . . . . . . . . . . . . | 13 times. |
| In the left hemisphere . . . . . . . . . . . . . | 9 " |
| In the vermiform processes . . . . . . . . . . | 5 " |
| In both hemispheres . . . . . . . . . . . . . . | 5 " |
| Not stated . . . . . . . . . . . . . . . . . . | 3 " |

These results agree with those of Macabian, who also found the prevalence of the tubercle deposits in the right hemisphere. Allo, however, found the deposits more frequently in the left hemisphere.

*Size.*—Tubercles of the cerebellum offer a great variance in regard to their size, and even in the same individual the extremes may occasionally be met. The size of solitary tubercles may range from that of a bean to that of an egg. Cases have been described in which whole hemispheres of the cerebellum have been transformed into tubercular growth. If the seat of a nodule be in a hemisphere or vermiform process, it chooses by rapid growth to take considerable dimensions. If, on the other hand, they appear in great numbers, are not disposed to conglomerate but retain their miliary character, they generally have the size of a pin's head. In general terms, the size is dependent upon the number—the greater the size the less the number, and vice versa.

From a clinical standpoint, the size of a nodule may be of great diagnostic importance. The pressure of large tubercles upon neighboring tissues may call forth characteristic symptoms, enabling the clinician to locate approximately its seat and size. Smaller growths may remain inert for long periods, producing no disturbance whatever, their presence being detected only at the autopsy.

*Number.*—As just mentioned, the number of tubercles depends more or less upon their size; miliary tubercles existing without number, while larger tubercles are much less numerous. In the case examined by me, there were present six to eight tumors, the size of a hazelnut, in the substance of the cerebellum. Cases have been described where eighteen to twenty nodules, the size of a bean, have been found in the cerebellum.

*Form.*—The form of tubercular growths is generally round or oval, which form depends more or less upon their concentric, laminated structure. If they appear at the periphery, and by rapid growth attain great size, they become flattened, owing to the hindrance offered by the bony parts. Large masses may present an uneven, irregular form, owing to the conglomeration of several nodules.

*Etiology.*—The origin of cerebral resp. cerebellar tubercles cannot be regarded as primary, and especially not when the tubercles appear in advanced life. To speak of a local tuberculosis of the cerebellum as one speaks of a lupus, a fungous growth of the joints, a tuberculosis of the testicle, scrofulous affections of the skin and glands, and other ulcerative processes, is unwarranted, although in several autopsies made by different observers, isolated tubercles of the cerebellum have been found.

External violence, especially injuries to the head, etc., never lead to the formation of cerebral tubercles, except in those cases where a predisposition to tuberculosis is present. In all cases, the development of cerebral-cerebellar tubercles must be regarded as secondary, having been preceded by a tubercular affection in some other organ.

In an analysis of eighty-two cases, Cubasch found five in which the first symptoms appeared immediately after an injury to the head. Fleischmann has seen the first symptoms arise in three cases after an intense fright. Cubasch has also observed the first symptoms after the cessation of eczemas of the head, and after the suppression of chronic discharges in scrofulous individuals.

The point of departure in such cases is generally found in cheesy degenerated lymphatic or bronchial glands, tuberculosis of the lungs, fungous affection of the points, caries of the bones, especially of the spine, tubercular affections of the testicles, prostate, bladder, etc.

*Age.*—In the great majority of cases, tubercular nodules of the cerebellum occur in childhood, and even in the earliest periods of life. Demme (seventeen years' report of the Berner hospital) found in a child two or three days old, a tubercular nodule, the size of a hazelnut, in the hemisphere of the cerebellum. Barthez and Rilliet have never observed cerebellar tubercles in children under three years of age. This assertion may be explained, however, by the fact that no child under five years was admitted into their hospital. Henoch, in reporting fourteen cases which he observed at the charitè (Berlin), found the ages to range from nine months to two years. Mauthner found, in thirty-two cases, the affection appear in children under three years of age, seventeen times. Fleischmann, in sixty-two cases, observed the affection seven times in children between eleven months and two years. In the thirty-seven cases which I gathered from recent literature, the age ranged from nine months to fifteen years. Pribram found, in forty-seven cases under fifteen years, the cerebellum fourteen times affected, while in seventeen cases which occurred in subjects over fifteen years, the cerebellum was found but three times to be the seat of the nodules.

From the above statistics it is evident that the great majority of cases occur between the second year and the age of puberty. In rare cases of chronic tuberculosis of adults, tubercles have been found in the cerebrum and cerebellum, but whose structures indicated in all probability that they were formed in early periods of life. Still rarer are the cases in which the nodules appear between the fiftieth and sixtieth year and beyond.

*Sex.*—As regards the sex, opinions and statistics differ. Barthez and Rilliet found that the majority of cases of tubercular nodules of the cerebellum occur in boys. Green, on the contrary, found that girls were more often affected. Cubasch found, in his analysis of seventy-seven cases, fifty-seven boys and twenty girls. Fleischmann found, in sixty-three cases, forty-four boys. In my analysis, I found that the boys were more predisposed than the girls. In fifty-three cases of tuberculosis of the encephalon, of which thirty-seven were limited to the cerebellum, I found twenty-eight boys and twenty-five girls.

*Termination.*—In the majority of cases where exitus letalis occurs, the nodules are in the stage of cheesy metamorphosis. In those cases where the life of the patient is spared for some time, and death occurs through an intercurrent disease, the regressive metamorphosis of the nodules may be observed in various places.

Cubasch, likewise Hasse, are of the opinion that the nodule may calcify. This process takes place where the growth of the tubercle is arrested in the stage of softening. "The soft pulpy masses undergo resorption, the contents thicken, forming a yellowish mealy mass, the nodule contracts, a deposition of calcareous salts takes place, leaving a hard stony irregular mass—in other words the nodule has been transformed into a calcareous body. Seeligmüller doubts that such a change is possible. Henoch has observed the calcification in two cases. In the first case he found in the tubercular nodules chalky concretions the size of a pea. In the second case he found in the nodules of the cerebellum several hard chalky masses.

Virchow has observed cases in which he found fine soft miliary granulations in the form of small light gray points in the neighborhood of the nodules. A closer examination showed them to be delicate new formations of connective tissue fibers, a sort of pseudo-membranous capsule forming the matrix of an exterior new growth. In these cases, the process may be considered as an encephalitis tuberculosa. In those cases where the fibrous elements of the neoplasm predominate, one may consider them as sclerosis tuberculosa.

Yet to be mentioned is a condition observed very often in tuberculosis of the cerebellum, as well as in an extended miliary tuberculosis of the cerebellum, viz., the presence of an exudation in the arachnoid cavity. The presence of the same is due, no doubt, to the inflammation of the pia.

More important, however, is the hydrocephalus ventriculorum which occurs very often in these cases. Barthez and Rilliet, and Barrier were the first ones to offer an explanation of this phenomena. Barthez and Rilliet, who observed the hydrocephalus only in the tuberculosis of the cerebellum, believe it to be due to pressure on the venæ Galeni. If the nodules be of sufficient size to produce compression of these veins, or to increase the arterial pressure in the cerebellum, a venous stasis must naturally follow, producing a transudation in the ventricles. Nodules situated in the superior vermiform process, or between the superior vermiform process and the tentorium cerebelli, may easily exert a pressure on the venæ Galeni, or on the sinus rectus.

Henoch regards the mechanical theory of hydrocephalus occurring in tubercolosis of the cerebellum as of secondary importance. He believes it to be due in many cases to an irritation to the tela choroidea superior and transmitted to the ependyma of the ventricles, thus producing a serious exudation.

5 Rue Rollin, Paris.

